# Bone Tissue Engineering for Dentistry and Orthopaedics

**DOI:** 10.1155/2014/241067

**Published:** 2014-11-26

**Authors:** Yin Xiao

**Affiliations:** ^1^Institute of Health and Biomedical Innovation, Queensland University of Technology, 60 Musk Avenue, Kelvin Grove, Brisbane, QLD 4059, Australia; ^2^Australia-China Centre for Tissue Engineering and Regenerative Medicine (ACCTERM), Brisbane, QLD 4059, Australia

Bone has a strong capacity for self-repair; however, conditions such as complex trauma, tumor, infection, and congenital disorders, which can cause large bone defects and resorption, often result in devastating deficits of dental and skeletal tissues. Clinically, this can lead to nonunion of bone and the loss of functional support to surrounding tissues, with the consequence of significant impact on the quality of life of patients. This is a challenging situation that typically requires bone grafting and complicated and expensive treatment strategies.

Currently the gold standard material for bone defect repair is autografts; however, donor site morbidity and limited supply prevent the wide application of this method. Allografts and xenografts can address the supply issue but face issues such as immune rejection and potential transmission of infectious diseases. In view of the limitations inherent with conventional bone graft strategies, tissue engineering represents a promising approach for bone repair and regeneration. Advances in tissue engineering have led to innovative scaffold designs, complemented by progress in the understanding of cell-based therapies and bioactive growth factor delivery.

Bone tissue engineering strategies have demonstrated that there is great potential to address the ever-pressing clinical need and have attracted attention from scientists, engineers, and clinicians worldwide over the past 25 years. This is reflected by the increasing interest shown by our readership and authors in this promising field and we are, therefore, pleased to present this special issue. In this issue, we have compiled fourteen exciting papers, including research articles and reviews that reflect the diversity of this fascinating subject and provide a better understanding of recent advances in the field of bone tissue engineering.

The fundamental concept of bone tissue engineering is to combine progenitor cells or osteogenically differentiated/mature cells (for osteogenesis) seeded onto biocompatible scaffolds and ideally in three-dimensional structures (for osteoconduction and vascular ingrowth), with appropriate growth factors (for osteoinduction) to generate functional bone structures. Effective cell-based therapies for bone tissue engineering typically employ the coordinated manipulation of cells and biologically active signaling molecules. Y. Wu et al. have demonstrated the potential use of temporomandibular joint derived synovial stem cells (TMJ-SDSCs) in TMJ disc repair and regeneration. Y. Zhou et al. report that a hypoxic microenvironment can maintain cell proliferation capacity, enhance pluripotency, and promote differentiation, indicating that effective cell isolation and expansion under hypoxic conditions may be a viable technique for autologous cell-based therapies. S. Tuan et al. present a review on the functional regulation of osteoblast lineage cells in health and osteoporosis, with an emphasis on the application of strontium and its role in regulating bone remodeling via its involvement in a number of pathways. B. Chen et al. have focused on the role of nuclear factor-*κ*B ligand (RANKL) in periodontal bone resorption and explored the factors involved in the regulation of the RANKL expression. Q. Zhang et al. provide an overview of the role of interleukin-10 (IL-10) in bone loss diseases and discuss the possibility of IL-10 adoption in the treatment of bone-related diseases, whereas K. Luo et al. show evidence that suggests that changes of the expression of cytokines and bone turnover markers in periodontium of ovariectomized rats can contribute to the damage of periodontal tissues.

Optimizing and refining the use of scaffolds is another important aspect for bone tissue engineering. Taking their cues from the extracellular matrix, C. Rentsch et al. have developed embroidered polycaprolactone-co-lactide (PCL) scaffolds that are coated with collagen/chondroitin sulphate and which can enhance* de novo* bone formation and be used as skull bone implants for large* in vivo* defects. M. Shi et al. have constructed multifunctional nanosized mesoporous bioactive glass/poly(lactic-co-glycolic acid) composite-coated CaSiO_3_ scaffolds that have improved mechanical strength, apatite-mineralization activity, cytocompatibility, and drug-delivery properties and which have promising applications in bone tissue engineering. G. Wu et al. have shown that the drug loading efficiency and release profile of bioactive scaffolds can be adjusted by changing the internal phase of the microparticles. This provides better understanding when fabricating multipurpose* in situ* drug releasing scaffolds for future clinical applications. X. Yu et al. report that the cellular responses to biomimetic calcium phosphate coatings are inferior to an alkaline-treated titanium surface, highlighting that substrate surface properties directly influence cell adhesion on different biomaterials.

With a firm focus on maintaining biomechanical properties, Z. Zhou et al. report that injection of hydrogel into the intervertebral discs can greatly restore the shock absorption of this tissue, suggesting that hydrogel injections may be a promising clinical approach to manage intervertebral disc degeneration. B. Lohberger et al. have evaluated the effects of cyclic tensile strain on the cell differentiation towards an osteogenic lineage, thereby contributing to a better understanding of strain-induced bone remodeling. E. Chung et al. further demonstrate that the combination of tensile and thermal stress conditioning over a short period has the potential to modify cellular performance and thus synergistically promotes bone regeneration. In order to provide scientific and empirical evidence for the clinical application of the polyaxial self-locking anatomical plate, W. Liang et al. have gathered geometrical data on the distal tibias and manufactured a variable locking screw trajectory to improve screw-plate stability through the design of a polyaxial self-locking anatomical plate.

Bone tissue engineering has become increasingly dependent on the emergence of innovations from all of these fields, even as they have continued to evolve independently. By gathering these papers in this issue, we seek to incorporate the diverse areas of research in order to reflect current trends. It is our hope that this will enrich our readers and the wide range of researchers in the field of bone tissue engineering for the application in orthopaedics and dentistry.

*Yin  Xiao*

